# Relocation of lower pole renal stones helps improve the stone-free rate during flexible ureteroscopy with a low complication rate

**DOI:** 10.1007/s00345-023-04703-6

**Published:** 2024-01-13

**Authors:** Ru Huang, Jian-chun Chen, Yong-qiang Zhou, Jin-jin Wang, Chu-chu Hui, Min-jun Jiang, Chen Xu

**Affiliations:** 1 Department of Urology, Suzhou Ninth Hospital Affiliated to Soochow University, Suzhou, 215200 China; 2 Department of Radiology, Suzhou Ninth Hospital Affiliated to Soochow University, Suzhou, 215200 China; 3 Department of Ultrasound, Suzhou Ninth Hospital Affiliated to Soochow University, Suzhou, 215200 China

**Keywords:** Relocation, Lower pole stone, FURS

## Abstract

**Objective:**

To compare the efficacy and safety of relocating the lower pole stones to a favorable pole during flexible ureteroscopy with in situ lithotripsy for the treatment of 10–20 mm lower pole stone (LPS).

**Methods:**

This study was a prospective analysis of patient outcomes who underwent an FURS procedure for the treatment of 10–20 mm lower pole renal stones from January 2020 to November 2022. The patients were randomized into a relocation group or in situ group. The LPSs were relocated into a calyx, during lithotripsy in the relocation group was performed, whereas the in situ group underwent FURS without relocation. All the procedures were performed by the same surgeon. The patients’ demographic data, stone characteristics, perioperative parameters and outcomes, stone-free rate (SFR), complications, and overall costs were assessed retrospectively.

**Results:**

A total of 90 patients were enrolled and analyzed in this study (45 per group) with no significant differences between the two groups in terms of age, gender, BMI, diabetes, hypertension, stone size, number, laterality, composition, and density. The mean operation time, total energy consumption, postoperative stay, and complications were similar between the groups. Both groups had similar SFR at 1 day postoperative follow-up (*p* = 0.091), while the relocation group achieved significantly higher SFR 3 months later (97.8% vs 84.4%, *p* = 0.026). The relocation group also had a significantly higher WisQol score than the in situ group (126.98 vs 110.18, *p* < 0.001).

**Conclusion:**

A satisfactory SFR with a relatively low complication rate was achieved by the relocation technique during the FURS procedure.

**Supplementary Information:**

The online version contains supplementary material available at 10.1007/s00345-023-04703-6.

## Introduction

Renal stones are one of the most common urologic diseases globally, with lower pole calculi accounting for almost 25% to 35% [1]. The best choice for lower pole stones (LPS) remains controversial, with several scholars recommending active surveillance [2, 3]. However, the Wisconsin Stone Quality of Life Questionnaire (WisQOL) score for most patients with LPS was poor [4] as patients were worried about the increase of the stone, as well as the renal colic caused by possible stone migration. The European Association of Urology (EAU) guidelines recommend Extracorporeal Shock Wave Lithotripsy (ESWL) as the first-line treatment for LPS < 10 mm, while Percutaneous Nephrolithotripsy (PCNL) is preferred for LPS > 20 mm [5]. For LPS of 10–20 mm, ESWL is also recommended, but the reported stone-free rate (SFR) is poor [6, 7]. PCNL could achieve excellent SFR but is limited by a greater risk of hemorrhage.

With improved technology, flexible ureteroscopy has become an attractive alternative for many urologists in the management of 10–20 mm LPS. Some researchers have recommended FURS for LPS due to its high success and low complication rates [1, 8–10]. However, the sharp infundibular–pelvic angle (IPA) resulted in the decreased SFR of FURS in LPS compared to upper or middle calyx stones. Therefore, several surgeons recommend relocating the LPS to a favorable calyx before lithotripsy to improve the SFR [1, 8, 11]. However, Shrestha et al. reported relocation of LPS followed by laser lithotripsy achieved similar SFR as in situ lithotripsy [12].

The current study compared the efficacy and safety of relocation during FURS with in situ lithotripsy for the treatment of 10–20 mm LPS. It was hypothesized that relocating LPS during FRUS could improve the SFR.

## Methods

A prospective randomized trial of 90 patients who underwent FURS with holmium laser lithotripsy for 10–20 mm LPS in our center was conducted from November 2020 to November 2022. The study protocol was approved by the local Institutional Review Board (Approval Number (KY2020-068-01)). All patients provided written informed consent before the operation. Necessary preoperative diagnostic procedures (medical history, serum creatinine and electrolytes, urine tests, sterile urine culture, 24-h urine electrolytes, and parathyroid hormone) were performed. Renal stones and kidney characteristics were assessed through low-dose abdominal none contrast computed tomography (NCCT) and plain X-rays of kidney–ureter–bladder (KUB). Patients with X-ray negative LPS, upper/middle pole stones, hyperparathyroidism, ureteral stricture, calyceal diverticular stones, medullary sponge kidney, and renal abnormalities (such as pelvic kidney or horseshoe kidney) were excluded. Data analysis included the patients’ demographics, stone characteristics, surgical details, perioperative outcomes, and SFR.

### FURS technique

The whole FURS procedure was performed by Doctor Xu (Doctor of Medicine, associate chief physician of the Urology Department) using an 8.4-Fr (Olympus, URF-V2, Japan) flexible ureteroscope. In our study, a double-J stent was routinely inserted into the target ureter under local anesthesia 2 weeks before FURS. Patients were placed in the dorsal lithotomy position under general anesthesia, and intravenous antibiotic according to the sterile urine culture was administered 30 min preoperatively in all cases (with negative urine culture, empirical use of antibiotics would be applied). Ureteroscopy was routinely performed using a semi-rigid ureteroscope (8F-9.8F, Richard Wolf GmbH, Knittlingen, Germany) before FURS in all patients, so that the preoperative double-J stent could be removed and two guide wires [a safe guide wire (HiWire, Cook Medical) and an operating guide wire (Sensor, Boston Science)] could be placed into the target renal pelvis. Thus, a hydrophilic-coated ureteral access sheath (Cook Medical, 12-14-Fr, Ireland) was inserted alongside the sensor wire under direct vision. LPS were treated using a 200 µm holmium laser fiber with a pulse energy of 0.8–1.0 J and pulse frequency of 10–30 Hz based on the volume and hardness of the stones. LPS were smashed directly in the in situ group. When LPS in the in situ group could not be managed by laser fiber, it was defined as lithotripsy failure and the patient was placed in the relocation group if the LPS could be displaced by a stone basket. In the relocation group, the LPS were removed to a favorable renal calyx (upper/middle poles) through a stone basket (TIPLESS Stone-extractor Nitinol, Reusable handle) before lithotripsy. Large-sized LPS that could not be engaged in the basket were first fragmented, and then, the fragments were relocated to the desired calyx for further lithotripsy. Basket retrieval was used for stones larger than 2 mm, while fragments ≤ 2 mm were left for spontaneous passage. A 4.7-Fr double-J stent was left in place in all cases. Stones were collected postoperatively to analyze the composition through Infrared Spectroscopy. Patients with LPS that could not be touched even when using the stone basket were excluded from this study and treated later with other appropriate treatments.

### Follow-up and statistical analysis

All patients underwent KUB radiography 1 day postoperatively to evaluate the primary SFR. The double-J stent was removed 2 weeks later by cystoscopy in outpatients. All patients obtained a KUB as well as a B-ultrasound 3 months after the first treatment. A subsequent procedure or other treatment modalities were chosen to help extract stones when faced with poor SFR. Stone-free status was defined as the absence of any stones in the KUB radiography and stone fragments ≤ 2 mm in B-ultrasound. Complications were graded according to the Clavien–Dindo classification system [13].

Statistical analysis was performed through SPSS statistical software version 27.0, with the data expressed as mean ± standard deviation (SD). The data were analyzed using the Chi-square test for categorical variables and the independent-sample T test for continuous variables. A *P* value < 0.05 was considered statistically significant.

## Results

A total of 90 patients with 10–20 mm LPS were enrolled in this study and randomized to two groups. The patients’, demographics and stone characteristics are outlined in Table 1, showing no significant difference between the two groups in terms of age, BMI, gender, diabetes, hypertension, and urine culture. Additionally, stone characteristics were also similar in terms of stone number, laterality, cumulative burden, composition, and density. The average diameter of the cumulative stone burden in the relocation and in situ groups was 13.2 mm and 13.7 mm, retrospectively.

**Table 1:** Comparison of patients, demographic and stone characteristics

Parameters	Relocation group (*n* = 45)	In situ group (*n* = 45)	*P* value
Age (years)	55.36 ± 13.08	53.09 ± 15.03	0.324
BMI (kg/m^2^)	24.56 ± 4.00	24.54 ± 3.13	0.226
*Diabetes*			0.581
Yes, *n* (%)	9 (20%)	7 (15.6%)	
No, *n* (%)	36 (80%)	38 (84.4%)	
*Hypertension*			0.667
Yes, *n* (%)	19 (42.2%)	17 (37.8%)	
No, *n* (%)	26 (57.8%)	28 (62.2%)	
*Urine Culture*			0.764
Positive, *n* (%)	7 (15.6%)	6 (13.3%)	
Negative, *n* (%)	38 (84.4%)	39 (86.7%)	
*Sex*			0.667
Male, *n* (%)	28 (62.2%)	26 (57.8%)	
Female, *n* (%)	17 (37.8%)	19 (42.2%)	
*Stone number*			0.822
single, *n* (%)	31 (68.9%)	30 (66.7%)	
multiple, *n* (%)	14 (31.1%)	15 (33.3%)	
*Stone laterality*			0.527
Right, *n* (%)	22 (48.9%)	25 (55.6%)	
Left, *n* (%)	23 (51.1%)	20 (44.4%)	
*Cumulative stone burden (mm)*			0.667
10–15 mm, *n* (%)	28 (62.2%)	26 (57.8%)	
15–20 mm, *n* (%)	17 (37.8%)	19 (42.2%)	
*Stone composition*			0.748
Calcium, *n* (%)	40 (88.9%)	39 (86.7%)	
non-calcium, *n* (%)	5 (11.1%)	6 (13.3%)	
*Stone density, *(Hu)	1121.36 ± 277.93	1102.56 ± 239.27	0.133

^*^Data represent means ± SD unless otherwise indicated

*BMI* Body Mass Index, *HU* Hounsfield Unit, *mm* millimeter, *SD* Standard Deviation

The perioperative parameters and outcomes of both groups are presented in Table 2. The average American Society of Anesthesiologists classification score was similar, with no significant differences in perioperative parameters including the operation time, total energy consumption, postoperative duration, and overall cost. Of note, the operative time for 10–15 mm LPS in the relocation group was shorter than in the in situ group, however, this was the opposite for the LPS of 15–20 mm, although not statistically significant (26.43 ± 11.13 vs 34.0 ± 16.34, *p* = 0.061, 59.41 ± 23.84 vs 47.63 ± 15.13, *p* = 0.065, respectively). The primary SFR in the relocation group and in situ group was 82.2% and 66.7%, respectively (*p* = 0.091). However, there was a significant difference three months later, as the SFR in the relocation group improved greatly compared to the in situ group (97.8% vs 84.4%, *p* = 0.026). Meanwhile, the WisQol score in the relocation group was higher than that of in situ group, with an excellent SFR (126.98 ± 10.13 vs 110.18 ± 23.95, *p* < 0.001).

**Table 2:** Comparison of perioperative parameters and outcomes between the groups

Parameters	Relocation group (*n* = 45)	In situ group (*n* = 45)	*P* value
ASA score^1^	1.24 ± 0.43	1.22 ± 0.42	0.623
Operation time^1^, (min)	38.89 ± 23.33	39.76 ± 17.08	0.064
10–15 mm	26.43 ± 11.13	34.0 ± 16.34	0.061
15–20 mm	59.41 ± 23.84	47.63 ± 15.13	0.065
Energy Used^1^, (KJ)	8.99 ± 0.94	8.84 ± 0.53	0.343
10–15 mm	8.41 ± 0.56	8.60 ± 0.47	0.190
15–20 mm	9.93 ± 0.61	9.16 ± 0.44	0.186
Postoperative stay^1^, days	2.91 ± 0.82	2.62 ± 0.61	0.877
Cost, (US Dollar)	3880.78 ± 353.26	3706.11 ± 290.66	0.303
Complication rate, *n* (%)	5 (11.1%)	4 (8.8%)	0.725
Grade I	1 (2.2%)	3 (6.7%)	–
Grade II	1 (2.2%)	1 (2.2%)	–
Grade IIIa	3 (6.7%)	–	–
SFR, *n* (%)			
Primary	37 (82.2%)	30 (66.7%)	0.091
3 months later	44 (97.8%)	38 (84.4%)	**0.026**
WisQOL score^1^	126.98 ± 10.13	110.18 ± 23.95	** < 0.001**

*ASA* American Society of Anesthesiologists, *min* minutes, *KJ* Kilojoule, *US* United State of America, *SFR* stone-free rate, *WisQOL* Wisconsin Stone Quality of Life Questionnaire

^1^Mean

Three patients in the in situ group were subsequently placed in the relocation group as the LPS could not be reached by the laser fiber, necessitating the use of the stone basket to relocate the LPS. The procedure was successful and we believed that this measure could effectively prevent the patients from accepting a second-stage procedure. The SFR of these three patients in situ group was defined as unclear.

According to the Clavien classification system, no serious intraoperative complications including minimal ureteral perforation occurred. Postoperative fever was the most common complication in both groups, with four patients administered indomethacin and recovering (Clavien grade I). Postoperative fever was found in two patients (1 in each group) who required antibiotics according to the urine or blood cultures (Clavien grade II). Two patients in the relocation group suffered a double-J-related fever, and thus, the double-J was immediately removed by cystoscopy under local anesthesia (Clavien grade IIIa). A new double-J stent was inserted under general anesthesia in one patient in the relocation group because of the migration of the double-J stent (Clavien grade IIIa).

## Discussion

The best treatment for 10–20 mm LPS remains controversial. The EAU guideline on urolithiasis recommends ESWL or endoscopy as the first-line treatment [5]. However, the SFR of ESWL is affected by many factors, such as the patient’s BMI and stone density. The reported success rate of ESWL for 10–20 mm LPS is 23% to 85% [6, 11, 14]. Recent data have proved that FURS could be an optimal approach for LPS [1, 8–11]. It is performed through the natural orifice, making it less invasive and safer than PCNL. The difficulty during FURS is mainly because of the narrow IPA and long lower pole infundibula [15]. The final SFR of LPS decreased, with the reduction life of the flexible ureteroscope due to intraoperative overbending. Several innovations have been applied to improve the SFR, including the ergonomics of the flexible ureteroscope, the use of access sheath with vacuum aspiration, changes in operation position, thinner laser fiber, and a stone basket [10, 16, 17]. In 2000, Kourambas et al. published their experience that relocating the LPS to a favorable calyx during FURS could improve the SFR but not significantly [18]. Subsequently, Schuster et al. reported that relocating the LPS could significantly improve the SFR [11]. Furthermore, Yaghoubian et al. shared a similar experience [8]. However, another study showed that relocating LPS during RIRS did not significantly improve SFR compared to in situ lithotripsy [12]. Therefore, we performed this prospective study to assess whether relocating LPS during FURS could improve the SFR.

In the present study, there was a significant difference in SFR between the relocation and in situ groups 3 months postoperatively. Three patients in the in situ group were reallocated to the relocation group as the stones could not be touched by the holmium laser. The stones were then relocated to the upper calyx through a stone basket, followed by a simple lithotripsy. Fragments in the upper or middle calyx may be easier to excrete but may move to other invisible calyxes during lithotripsy in situ. Additionally, certain activities such as jumping and handstands are quite difficult for elderly or obese patients, thereby increasing the risk of stone fragments residue.

Several surgical positions have been applied to improve the SFR of LPS. Zhong et al. recommended FURS in the lateral position for LPS, with a satisfactory SFR 1-month follow-up postoperatively [10]. Liaw et al. reported that the T-tilt patient position was associated with higher SFR [19]. In the present study, the untraditional dorsal lithotomy position (30°Trendelenburg) was used to access LPS fragments in the upper or middle pole more easily during the surgery. Meanwhile, a 4.7-Fr double-J stent was inserted into the target renal pelvis preoperatively, as the dilation of the ureter may be helpful to the stone fragments extraction, as well as decrease the risk of ureter injury caused by the access sheath.

In the current series, X-ray negative stones were excluded and KUB was used to evaluate SFR 1 day postoperatively. B-ultrasound was used to assess SFR 3 months later. The overall SFR was 91.1%, with the SFR in the relocation group significantly improved compared to the in situ group. Golomb et al. reported an excellent SFR with the displacement of LPS during RIRS, similar to the present study [1]. Another study reported the SFR of LPS via RIRS was 85.7% [10], which might be related to the larger stone size and the short 4-week follow-up. The technique of lithotripsy relied on the stone’s characteristics. Typically, we chose “Dusting” first and fragmentation later, as the stone core was hard and difficult to be dusted. Basket retrieval was routinely performed to help relocate the LPS and improve the immediate SFR in both groups. In our series, most LPS were smaller than 15 mm, and the dilation via the double-J stent before FURS may also contribute to the satisfactory final SFR. All the cases with fragments residue in our study were concentrated on stone burden larger than 15 mm. For such cases, a curved end suctioning access sheath might be an optimal choice, and a PCNL technique, such as super-mini percutaneous nephrolithotripsy (SMP) or ultra-mini PCNL [20, 21], is also a good option for LPS with hydrocalycosis.

Regarding operative time and total laser energy consumption, Schuster et al. reported a significantly longer operative time in the relocation group [11], whereas Yaghoubian et al. reported that the operative time was slightly increased in the relocation group, as well as the laser energy consumption, although with no statistical significance [8]. A similar result was achieved by Shrestha et al. [12]. There was also no significant difference between the two groups in our study, due to the improved ergonomics and the popularization of flexible ureteroscopy in the last 2 decades.

The main purpose of stone treatment modalities is to achieve maximum SFR with minimum complications. Our overall complication rate was 10%, which is acceptable and comparable to published data [8–12]. Postoperative fever was the most common complication in our study but no urine-related sepsis or ureteral injury was observed. Additionally, no second-stage procedure was required in either group.

WisQOL is a reliable tool to evaluate the quality of life for patients with renal stones [4, 22–25]. In our study, patients in the relocation group had a significantly higher WisQOL score than in the in situ group, postoperatively, probably due to the higher SFR in the relocation group. The overall cost of the procedure was not increased with the use of a stone basket and the double-J stent placement before FURS. We used a reusable flexible ureteroscope instead of a single-use scope. Furthermore, the use of the basket and the ureter dilation procedure may help increase the postoperative fragments extraction and the success rate of the one-stage FURS procedure.

This study has several limitations. First, the study sample was small and a further prospective randomized-controlled trial with more cases is required to confirm our findings. Second, KUB and B-ultrasound were used to assess the SFR of LPS instead of NCCT postoperatively, although we excluded the X-ray negative LPS, NCCT is still the most sensitive image modality to assess the SFR. Finally, we did not measure the infundibular characteristics in all patients, so a longer term follow-up may be needed to calculate the SFR in both groups.

## Conclusion

The final SFR was significantly higher in the relocation group with an acceptable low complication rate compared to the in situ group, indicating that relocating the LPS to a favorable calyx during FURS is effective and safe for the treatment of 10–20 mm LPS.

## Supplementary Information

Below is the link to the electronic supplementary material.

Supplementary file1 (PDF 2563 KB)

Supplementary file2 (XLS 34 KB)

Supplementary file3 (XLS 33 KB)

## Data Availability

The authors confirm that the data supporting the findings of this study are available within the supplementary files of the article.
