# Supplementary material for: Relocation of lower pole renal stones helps improve the stone-free rate during flexible ureteroscopy with a low complication rate
Source: World J Urol. 2024 Jan 13;42(1):30. doi: 10.1007/s00345-023-04703-6 (PMC10787685; doi:10.1007/s00345-023-04703-6)
Supplement: Supplementary file 1 — Supplementary file1 (PDF 2563 KB) [file 345_2023_4703_MOESM1_ESM.pdf]

医学伦理审查报告

批件号：KY2020-068-01

苏州市第九人民医院拟开展“ Relocation of lower pole renal stones helps improve the stone free rate during flexible ureteroscopy procedure with low complication rate”科研工作，该项目涉及人体实验，我院伦理委员会对该项目相关医学伦理学问题进行了审查。

拟申报项目信息：

项目类型：自筹项目

研究项目名称：Relocation of lower pole renal stones helps improve the stone free rate during flexible ureteroscopy procedure with low complication rate

承担单位：苏州市第九人民医院

项目负责人：蒋民军(主任医师)　徐辰（副主任医师）

研究起止日期：2020 年 11 月-2022 年 11 月

涉及人体实验的主要内容：

我们对 2020 年 11 月至 2022 年 11 月在我们中心接受 10-20mm 肾脏下盏结石治疗的患者进行了一项前瞻性随机试验。所有患者术前均签署知情同意书。对所有入组患者进行了必要的术前诊断程序（病史、血清肌酐和电解质、尿液测试、无菌尿培养、24 小时尿液电解质和甲状旁腺激素）。通过低剂量腹部无对比计算机断层扫描（NCCT）和肾输尿管膀胱平片（KUB）评估肾结石和肾脏特征，CT 也可用静脉肾盂造影替代。X 线阴性 LPS、肾脏上/中盏结石、甲状旁腺功能亢进、输尿管狭窄、肾盏憩室结石、髓海绵质肾、肾异常（如盆腔肾或马蹄肾）的患者被排除在本研究之外。收集患者的人口统计学特征、结石特征、手术细节、围手术期结局和术后结石清除率。

审查评议意见

经我院伦理委员会审议，该研究的实验设计和实施方案充分考虑了安全性和公平性原则，研究内容不构成对受试者的伤害和风险，受试者的招募将完全基于自愿和知情同意原则，并尽最大限度保护受试者的隐私，研究内容和结果不存在利益冲突。

结论

该研究中，受试者权力和利益得到充分保护，对受试者不存在潜在风险. 同意该项研究的现场工作按计划进行。

苏州市第九人民医院

临床医学研究伦理委员会

2020 年 10 月 30 日
